# Editorial: Micro/nano optical devices for biosensing and cellular analysis

**DOI:** 10.3389/fbioe.2022.979707

**Published:** 2022-08-15

**Authors:** Srabani Kar, Moeto Nagai, Tuhin Subhra Santra

**Affiliations:** ^1^ Department of Engineering Design, Indian Institute of Technology Madras, Chennai, India; ^2^ Department of Electrical Engineering, University of Cambridge, Cambridge, United Kingdom; ^3^ Department of Mechanical Engineering, Toyohashi University of Technology, Toyohashi, Japan

**Keywords:** optical biosensors, plasmonic biosensors, optoelectronic tweezers, dielectrophoresis, cancer biomarkers

Recent advancements in micro technologies and nanotechnologies integrated with chemical, electrical, optical, biological, and biomedical engineering, generating a new era to developed micro/nanoscale optical devices for diverse types of biomolecular sensing and cellular analysis.

For biosensing and cellular analysis, optical devices such as metamaterial, metallic and semiconducting nanostructures, and plasmonic devices have sparked a lot of research interest. They exhibit great sensitivity and robustness and can potentially fit on a single chip. Because of their significant capability to control light at subwavelength scales, metamaterial and plasmonic devices have drawn enormous attention for various applications beyond conventional techniques. The efficiency of these devices as optical biosensors has proven to be worthwhile in detecting and manipulating various biomolecules.

For cellular analysis in a complex micro-/nanofluidic environment, micro/nanofluidic optical devices have proven to be powerful techniques and inevitable tools, with minimum sample consumption and less contamination than bulk analysis. The ability to manipulate and detect biomolecules, bio-samples and reagents at the micro/nano scale level can satisfy the needs of diverse biosensing and cellular analysis. The devices not only perform precise cellular manipulation, separation, isolation, and lysis but can also provide cellular electrical, mechanical and optical characterization.

This Research Topic of Frontiers of Bioengineering and Biotechnology entitled “*Micro/Nano Optical Devices for Biosensing and Cellular Analysis*” covers the recent advancements in biosensing and cellular analysis using different approaches.

Broadly neutralising antibodies (bnAbs) against human coronavirus (HCoV) is a vital tool for future coronavirus pandemic planning. Sypabekova et al. suggested utilizing state-of-art fiber optic biosensors to identify broadly neutralizing antibodies (bnAbs). As diagnostic and environmental sensing tools, discovered bnAbs should be able to detect HCoVs after being mounted onto fiber-optic biosensors. These bnAbs have different therapeutic and preventative applications for passive immunisation and develop a universal vaccine. The author emphasised the capability of the HCoV spike-functionalized biosensor to identify bnAbs.

The gold nano-particles (AuNPs) have significant promise in the biomedical field due to their chemical stability, excellent biocompatibility, simple synthesis method, excellent optical properties related to surface plasmon resonance, and minimal toxicity. Tai et al. reviewed AuNPs based optical biosensors for cancer biomarkers proteins. The AuNPs can enhance sensitivity and improve the device’s resolution, contrast, and specificity. These AuNPs may detect the protein biomarkers at a low threshold. The author reviewed AuNPs synthesis and related optical biosensors on the principle of localized surface plasmon resonance (LSPR), luminescence, and surface-enhanced Raman spectroscopy (SERS).

Siao et al. experimented on the hydrogen peroxide (H_2_O_2_) secretion from living cells. Due to mitochondrial electron transport, the reactive oxygen species (ROS) are produced within cells. Excessive ROS can cause cell necrosis or apoptosis. One vital ROS, H_2_O_2_, performs chemical processes in organisms and can control homeostasis. In this instance, the author employed a hydrogel-based substrate prepared by mixing gold nano-rods (AuNRs) to segregate large molecules (such as proteins) and directly detect H_2_O_2_ released from living cells. It was a LSPR based optical detection method. The author demonstrated how cultural forms of the cells considerably impact their H_2_O_2_ secretion.

Vila et al. proposed disposable polymeric nanostructured as a plasmonic biosensor to monitor adhesion and proliferation of human retinal pigmented cells (ARPE-19) under variedmedia conditions (0 and 2% of FBS). Compared to 0% FBS, they noticed a gradual rise in the plasmonic band displacement with time (1, 3, and 5 h) at 2% condition. Thus, the proposed biosensing technique is a straightforward tool to investigate the activity of live cells under culture conditions in real-time.

Polasky et al. studied the effects of superparamagnetic iron oxide nanoparticles (SPIONs) on THP-1 monocytes and monocytes-derived macrophages. The SPION_S_ have potential uses for 3-D imaging with great resolution, sensitivity, and speed. The dextran-coated SPIONs were incubated with THP-1 monocytes, macrophages (monocyte-derived), and peripheral blood monocytes. The authors showed that monocytes and macrophages quickly ingested SPIONs, with no corresponding decline in cell viability.

Vasudevan et al. proposed a simple photoluminescence-based assay for acyl-homoserine lactones (AHL) detection employing cysteamine-capped titanium oxide (TiO_2_) nanoparticles. The C4-HSL and 3-oxo-C12 HSL AHL molecules of *Pseudomonas aeruginosa*, a opportunistic human pathogen, were used to verify the bioassay. The authors demonstrated the fluctuation of the photoluminescence intensity associated to the oxygen defect state of TiO_2_, indicating its potential applications for biosensing.

Liang et al. employed optoelectronic Tweezers (OETs) to manipulate and assemble the micro-spirals into multiple microstructures. This technique provides precise manipulation of micro-bio-particles by applying light patterns. The simulation results indicated that a local light pattern by OETs can control micro-spirulina in real-time. It can be easily put into microstructures, including T-shape circuits, micro-coil devices, and link levers. With its ability to control flow, electric, and magnetic fields, the micro-spiral offers various applications, including drug delivery, material deformation, and micro-electronic tools.

Although optically induced dielectrophoresis (ODEP) is a well-known and effective method for manipulating cells, it is constrained by the need for low conductivity solutions. Chu et al. investigated to mitigate the impacts of low-conductivity solutions. They separately assessed cell viability in ODEP assays using sucrose solution and BSA-supplemented sucrose, finally revealing superior performance in the latter condition. The major purpose of this study was to do further basic research in order to improve background solutions in ODEP investigations.

In this editorial, the suggested micro/nano optical biosensing and cell manipulation techniques have demonstrated their potency and effectiveness to detect biomarkers, biomolecules, etc., as well as to analyse and monitor the activity of live cells under various circumferential conditions. This Research Topic might be viewed as prospective methodologies, albeit they would still need to overcome the challenges of commercialization and diagnostics applications.

